# Deletion of *Plasmodium falciparum ubc13* increases parasite sensitivity to the mutagen, methyl methanesulfonate and dihydroartemisinin

**DOI:** 10.1038/s41598-021-01267-6

**Published:** 2021-11-08

**Authors:** Supawadee Maneekesorn, Ellen Knuepfer, Judith L. Green, Parichat Prommana, Chairat Uthaipibull, Somdet Srichairatanakool, Anthony A. Holder

**Affiliations:** 1 Department of Biochemistry, Faculty of Medicine, Chiang Mai University, Chiang Mai, 50200 Thailand; 2 Malaria Parasitology Laboratory, The Francis Crick Institute, 1 Midland Road, London, NW1 1AT UK; 3 Medical Molecular Biotechnology Research Group, National Center for Genetic Engineering and Biotechnology (BIOTEC), 113 Thailand Science Park, Phahonyothin Road, Khlong Nueng, Khlong Luang, 12120 Pathum Thani Thailand; 4 Molecular and Cellular Parasitology Laboratory, Department of Pathobiology and Population Sciences, The Royal Veterinary College, Hawkshead Lane, Hatfield, AL9 7TA UK; 5 Thailand Center of Excellence for Life Sciences (TCELS), Phayathai, 10400 Bangkok Thailand

**Keywords:** Biochemistry, Microbiology, Diseases, Pathogenesis

## Abstract

The inducible Di-Cre system was used to delete the putative ubiquitin-conjugating enzyme 13 gene (*ubc13)* of *Plasmodium falciparum* to study its role in ubiquitylation and the functional consequence during the parasite asexual blood stage*.* Deletion resulted in a significant reduction of parasite growth in vitro*,* reduced ubiquitylation of the Lys63 residue of ubiquitin attached to protein substrates, and an increased sensitivity of the parasite to both the mutagen, methyl methanesulfonate and the antimalarial drug dihydroartemisinin (DHA), but not chloroquine. The parasite was also sensitive to the UBC13 inhibitor NSC697923. The data suggest that this gene does code for an ubiquitin conjugating enzyme responsible for K63 ubiquitylation, which is important in DNA repair pathways as was previously demonstrated in other organisms. The increased parasite sensitivity to DHA in the absence of *ubc13* function indicates that DHA may act primarily through this pathway and that inhibitors of UBC13 may both enhance the efficacy of this antimalarial drug and directly inhibit parasite growth.

## Introduction

*Plasmodium falciparum*, the parasite responsible for the most severe malaria and one of the leading causes of infant mortality in Africa, has a complex multi-stage life cycle, with sexual reproduction in the *Anopheles* mosquito and asexual proliferative stages within hepatocytes and red blood cells (RBCs) of a human host. During the asexual blood phase of its life cycle, every ~ 48 h the parasite invades new RBCs, grows and multiplies within them, developing through ring, trophozoite and schizont stages to produce extracellular merozoites that invade new RBCs. Because of its high replication rate and the accompanying rapid cell transformations, it is likely that the reuse of resources, the control of protein quality, and accurate DNA synthesis machinery are important features of progression through the life cycle^1^.

The Ubiquitin–Proteasome System (UPS) is a highly regulated cellular mechanism of protein turnover with critical roles in protein degradation, signal transduction, cell cycle progression, transcriptional regulation and DNA synthesis and repair^2^. Protein ubiquitylation is a cascade of reactions carried out by three classes of enzymes: ubiquitin-activating enzymes (E1s), ubiquitin-conjugating enzymes (E2s) and ubiquitin ligases (E3s). E1 uses AMP to activate the C-terminus of ubiquitin, forming a covalent thioester to an E1 cysteine, and then in a transthiolation reaction transfers the activated ubiquitin to a cysteine side chain of an E2. Either directly, or facilitated by the involvement of an E3, the ubiquitin is transferred again and covalently conjugated to specific protein substrates, usually through a lysine residue. However, some E2s can transfer ubiquitin directly to the substrate N-terminal amino group or to Lys, Cys, Ser and Thr side chains, thereby playing a role in where and how a target is modified to form diverse ubiquitin linkages^3,4^. Polyubiquitin (poly-Ub) chains are often added to substrates; these chains are formed by ubiquitylation of ubiquitin through one of its seven lysine residues (K6, K11, K27, K29, K33, K48, and K63)^5^. The nature of this linkage in poly-Ub chains is very important for both proteasome-dependent and proteasome-independent functions. For example, in well-studied model cells, poly-Ub chains containing K48 and K11 linkages target substrate proteins for degradation by the 26S proteasome, while poly-Ub chains containing K63 linkages have other roles in a variety of cellular processes, including DNA repair, kinase activation, and vesicle trafficking^5,6^.

In the *P. falciparum* genome, many genes are annotated as coding for UPS–related proteins^7,8^, including ubiquitin-activating enzyme, PfUba1, (PF3D7_1225800) and at least eight potential E2s. One of these E2 genes (PF3D7_0527100) encodes putative ubiquitin-conjugating enzyme 13 (UBC13), which catalyses the formation of K63-linked polyubiquitin chains, as shown in a variety of organisms, including human (*Homo sapiens* [Hs]), rodent (*Mus musculus)*, yeast (*Saccharomyces cerevisiae* [Sc]*)*, fly (*Drosophila melanogaster),* plant (*Arabidopsis thaliana*), worm *(Caenorhabditis elegans)* and trypanosome (*Leishmania spp.)*^9–16^. UBC13 functions as part of a complex with an E2-related protein that lacks the active site cysteine, and is called Ubs variant (UEV). Different UEVs such as MMS2 or UEV1A function together with UBC13 to catalyze polyubiquitylation in different cellular processes. In *P. falciparum,* there is one E2-like protein (encoded by PF3D7_0305700) lacking the cysteine and with high similarity to ScMMS2. The mutation of *ubc13* and *mms2* genes in *S. cerevisiae* results in greater sensitivity than wild type cells to UV exposure and also greater sensitivity to treatment with the alkylating agent methyl methanesulfonate (MMS) that induces DNA damage. *ubc13* and *mms2* genes are epistatic and belong to the same DNA-repair pathway^12^. In *Arabidopsis*, mutations of UBC13 result in increased sensitivity to stress conditions, such as high salt, oxidative stress, and treatment with an abscisic acid (ABA) analogue; UBC13 (and two other E2s, UBC7 and UBC14) are important in the response of plants to multiple stress conditions^17^. HsUBC13 functions together with its two cofactors, Uev1A (UBE2V1) and Mms2 (UBEV2), to promote the formation of K63 poly-Ub chains, which leads to either NF-κB activation or recruitment of repair proteins to DNA lesions, respectively^9,18,19^. UBC13 also regulates other cellular processes, including nuclear localization of the tumour suppressor p53 protein and MAPK activation^20,21^. However, neither UBC13 nor UEV alone can promote K63-linked poly-Ub chain formation^22,23^.

PfUBC13 (PF3D7_0527100) is a 152-residue protein that was first identified as a substrate of the *P. falciparum* Protein Kinase 9 (PfPK9), an essential kinase of *P. falciparum* that does not appear to be a member of any known eukaryotic kinase family^24–26^. It was reported that Ser106 of PfUBC13 is phosphorylated by PfPK9, resulting in suppression of UBC13 activity^27^. However, in contrast, inhibition of PfPK9 decreased PfUBC13 activity and resulted in the reduction of K63-linked polyubiquitylation^24^. Both of these studies suggest that phosphorylation of PfUBC13 is important in controlling its function. The structures of recombinant putative PfUBC13 monomer and PfUBC13 in complex with Pf MMS2/UEV1 (PF3D7_0305700) have been determined (https://www.rcsb.org/structure/2r0j and https://www.rcsb.org/structure/3e95, respectively). NSC697923 (2-[(4-methylphenyl)sulfonyl]-5-nitrofuran) is a small molecule covalent inhibitor of UBC13^28^, which also inhibits the growth of *P. falciparum*, *in vitro*^29^, although its mode of action against the parasite has not been established. Since the putative *Pfubc13* is essential for *P. falciparum* asexual blood stage development^26^, it is possible that this protein might be a new target for anti-malarial drug development.

In this study, we aimed to identify the role of UBC13 in *P. falciparum* using an inducible DiCre-recombinase-based gene knockout approach. Following *Pfubc13* inducible gene knockout (iKO) there was decreased K63 linked poly-ubiquitylation and a growth defect. Furthermore the *ubc13-iKO* parasite showed higher sensitivity to MMS and to dihydroartemisinin (DHA), an antimalarial drug that also affects DNA repair^30,31^. Parasite treatment with NSC697923 caused a growth defect similar to that of the *ubc13-iKO*. These results suggest that *Pfubc13* is involved in K63 polyubiquitylation, is essential for the proper growth of *P. falciparum* and is important for the correct function of the DNA repair system. Inhibitors of UBC13 are likely to inhibit parasite growth and also enhance the activity of DHA and similar artemisinin-derived compounds against the parasite.

## Results

### Comparison of the putative *P. falciparum* UBC13 with the human enzyme

**Figure 1:**
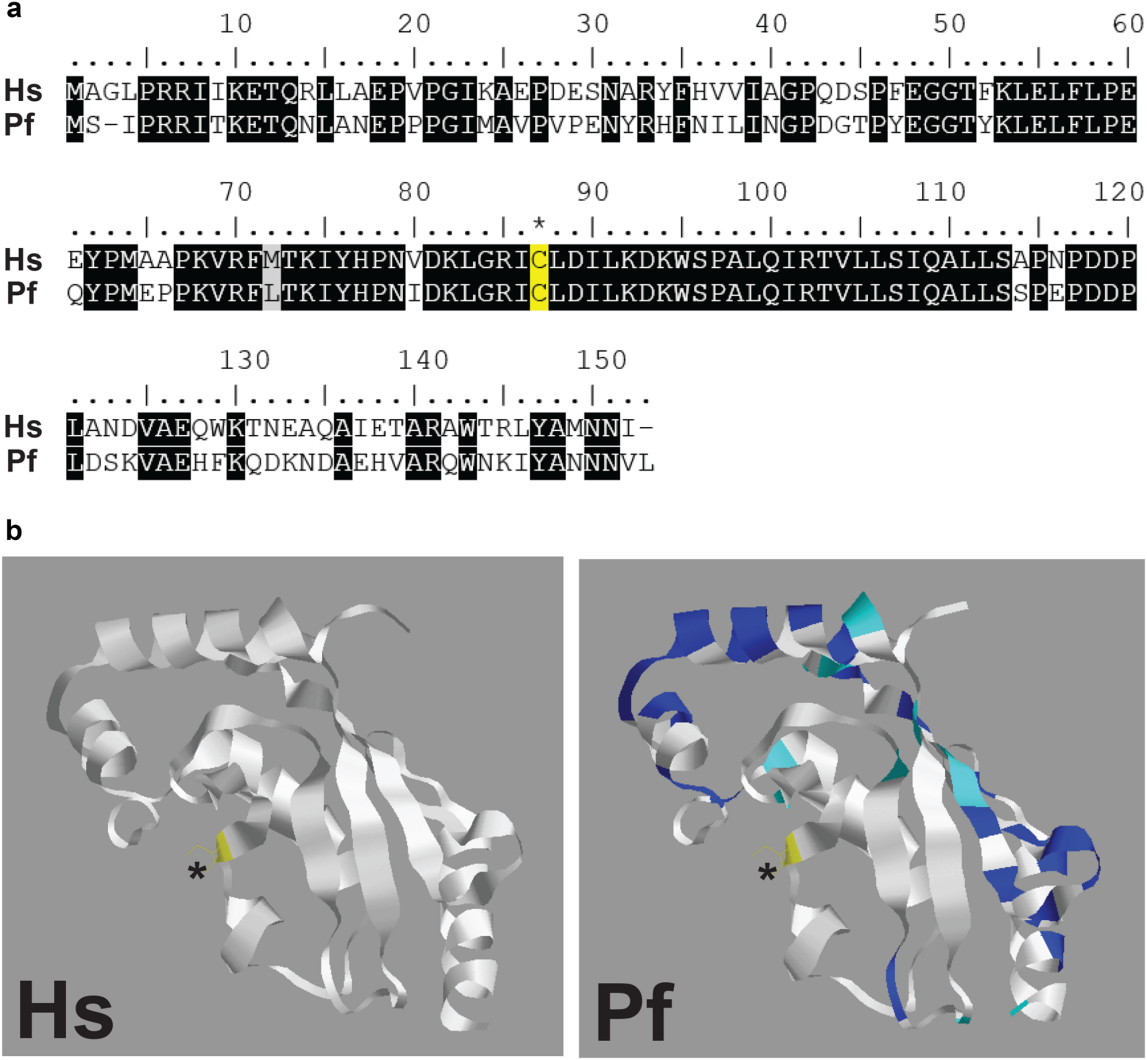
Structural comparison of HsUBC13 and the putative PfUBC13. (**a**) *Homo sapiens* (Hs)UBC13 and *Plasmodium falciparum* (Pf)UBC13 amino acid sequences are aligned, with identical residues in the two sequences highlighted in black. The active site cysteine residue that binds activated ubiquitin covalently and reacts with NSC697923 in human UBC13 is highlighted in yellow and marked with an asterisk. (**b**) Three- dimensional structures with the active site cysteine highlighted in yellow and marked with an asterisk. In the PfUBC13 structure, amino acid residues where the two sequences differ are highlighted in blue, or in cyan if the substitution is conservative.

An alignment of the HsUBC13 and putative PfUBC13 revealed that the 152-residue protein coded by PF3D7_0527100 is orthologous to HsUBC13 with 68% amino acid sequence identity (Fig. 1a). Most of the sequence differences are located away from the active site cysteine (Fig. 1b).

### Generation of an inducible *ubc13* knockout in *P. falciparum*

We used CRISPR-Cas9 to modify the 3’ end of the putative *ubc13* gene (PF3D7_0527100), inserting a loxP element in the fourth and final intron, inserting a recodonised final exon and placing a loxP site after the stop codon in the *ubc13* sequence (Supplementary Fig. 1a). The changes were made in the II-3 parasite line, which expresses functional DiCre recombinase activity following rapamycin addition^32^. After transfection, the parasite population was screened by PCR for DNA integration using diagnostic primer pairs P1/P2, P1/P4, P3/P4, P1/P6 and P5/P4 (Supplementary Fig. 1b, Supplementary Table S1) and products from both modified and unmodified locus were detected (Supplementary Fig. 1c). Parasites were cloned by limiting dilution and integration was examined in individual clones; for recombinant clones, primers P1/P6 and P5/P4, produced bands of 589 and 550 bp, respectively, diagnostic of integration and no product was obtained from primer pairs P1/P2, and P3/P4 indicating the absence of unmodified locus (Supplementary Fig. 1c). The parasite clone 2E was used in subsequent experiments.

### Induced excision of *ubc13* shows that the gene is essential for the growth and survival of *P. falciparum* asexual blood stage

To truncate *Pfubc13* and abolish expression of functional protein, we used a synchronized population of cloned parasites and at 4 h after invasion treated it with rapamycin or with DMSO alone (the control) for 24 h. Excision of the floxed DNA was monitored by PCR amplification with primer pair P1 and P4 (Supplementary Fig. 1d). For the control (DMSO-treated) sample a 1280 bp band was amplified, indicating no excision, and from the rapamycin-treated sample a 975 bp band was observed, indicating quantitative excision of the floxed DNA (Supplementary Fig. 1e). This result indicates that by the late trophozoite stage at 28 h after invasion, only a truncated *ubc13* gene remained following rapamycin-treatment.

**Figure 2:**
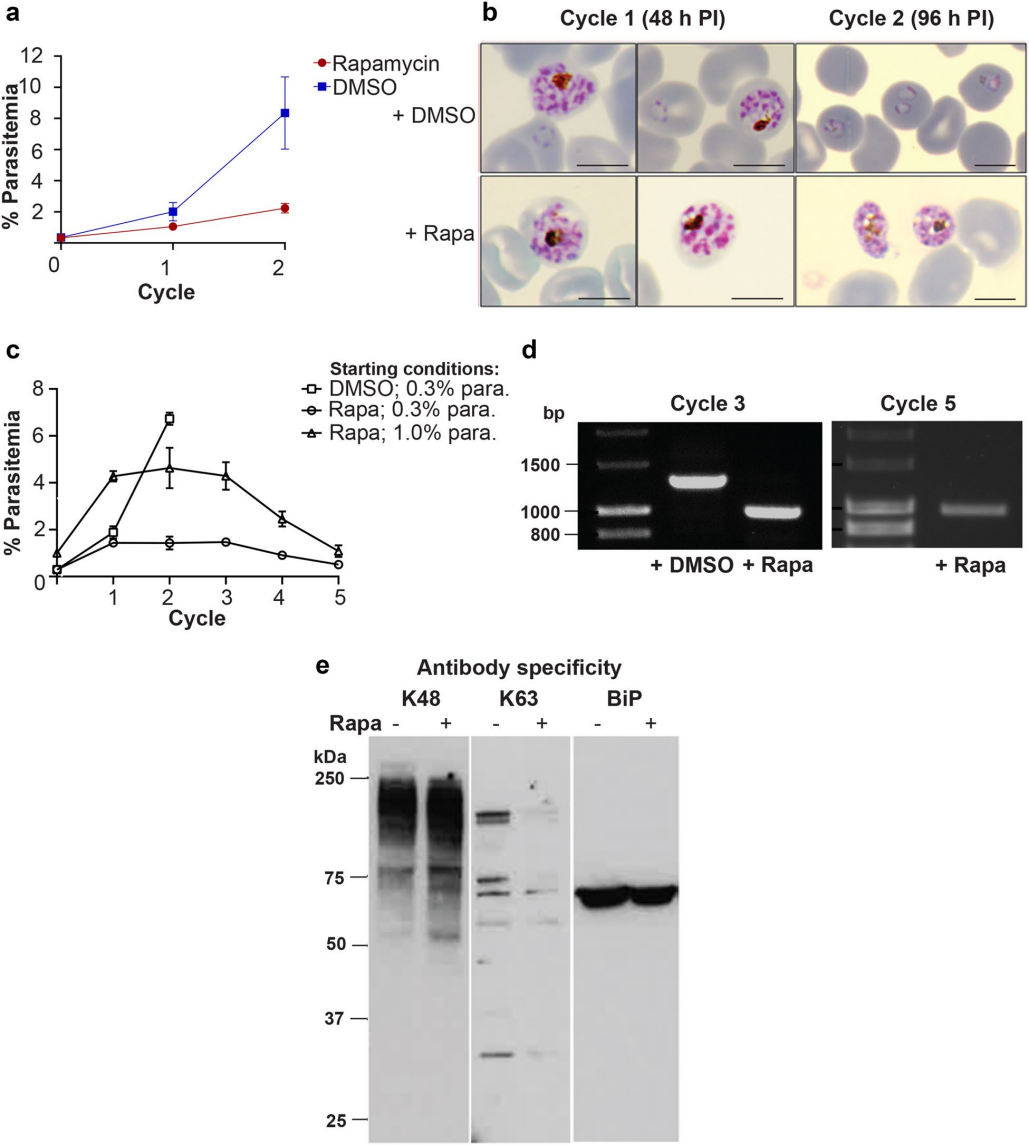
Effect of an inducible *Pfubc13* knockout in *P. falciparum* on parasite growth and ubiquitin ubiquitylation (**a**) Growth over two cycles of intraerythrocytic development for parasites treated with either DMSO (control) or rapamycin to induce Di-Cre mediated excision within *ubc13*. A synchronised parasite population (0.2% parasitemia) was treated with either DMSO or rapamycin for 24 h; parasitemia was measured by FACS analysis. Data are shown as mean ± standard error of the mean (SEM) from triplicate experiments performed in duplicate. (**b**) Analysis of parasites by light microscopy of Giemsa-stained thin smears, from parasites treated with either DMSO or rapamycin. At 48 h post invasion (PI), schizonts and ring stages were visible in DMSO treated cultures and there were fewer ring stages in the rapamycin treated cultures. By 96 h PI, second cycle ring stages were visible in DMSO-treated cultures and morphologically abnormal late stage parasites were predominant in rapamycin-treated cultures. Scale bar is 5 µm. (**c**) Parasitemia over up to 5 cycles of intraerythrocytic growth. The control (DMSO-treated) parasites were monitored for only two cycles; starting parasitemia, 0.3%. The rapamycin-treated parasites were monitored over five cycles; starting parasitema, 0.3 and 1%. The data shown are mean ± SEM from duplicate experiments. (**d**) The parasite populations were monitored by PCR amplification with primers P1/P4 (see Supplementary Fig. 1) at cycle 3 and cycle 5; no parasites without excision were detected in the rapamycin treated sample**. **(**e**) Analysis of schizont lysates by immunoblotting with antibodies specific for either Lys48 or Lys63 ubiquitylation of ubiquitin. Equal amounts of protein from rapamycin-treated or –untreated parasites at 44 h PI were resolved by SDS-PAGE, transferred to nitrocellulose and then probed with antibodies specific for either K48 or K63 ubiquitin linkages, or antibodies specific for BiP as a loading control. Full length gels and immunoblots are presented in Supplementary Figs. 3 and 4.

We next investigated whether functional *ubc13* is essential for parasite growth and survival. Parasites were treated at 2 h post invasion with rapamycin or DMSO for 24 h and then further incubated for either one (48 h) or two (96 h) cell cycles and analysed by flow cytometry (FACS) and examination of Giemsa-stained thin blood smears by microscopy. At 48 h the parasitemia had increased in both samples (Fig. 2a) but was significantly less (p < 0.008) following rapamycin treatment, with fewer new ring-stage forms resulting from merozoite release and reinvasion (Fig. 2b). There was a clear difference in the parasitemia of the control and rapamycin-treated parasites at the second cycle (p < 0.0002) (Fig. 2a). At 96 h most of the parasites were at the ring stage of development in the control cultures, whereas the rapamycin-treated parasites were largely at the schizont stage, and looking very unhealthy on Giemsa-stained smears. Little increase in parasitemia had occurred overall (Fig. 2b).

Further development of the rapamycin-treated parasite was monitored for up to five cycles (Fig. 2c). Parasites were still detectable in the culture although the parasitemia gradually declined. Analysis by PCR of DNA collected at cycles 3 and 5 confirmed that parasites contained the truncated gene, rather than representing parasites in which the gene was still intact (Fig. 2d). After the second cycle the rapamycin-treated parasites lost synchrony of development and some visible abnormalities were present in late stages, such as pyknotic and deformed cells (Supplementary Fig. 2). These results indicate that *ubc13* is not absolutely essential for completion of the first growth cycle following rapamycin treatment, perhaps because of residual active UBC13 protein. However, for the second and subsequent cycles the absence of growth and decrease in parasitemia, coupled with the abnormal morphology of many parasites, indicate that UBC13 is essential for asexual blood stage growth of *P. falciparum*.

### *ubc13* deletion affects K63 polyubiquitylation in the parasite

We examined whether or not deletion of the putative *ubc13* affected polyubiquitylation in the parasite. We used two antibodies, one specific for ubiquitin ubiquitylation at lysine-48 (K48) and the other for ubiquitin ubiquitylation at lysine-63 (K63). Parasites were treated with rapamycin or DMSO as described above and then extracts were prepared from schizonts for western blotting with the two antibodies (Fig. 2e). In a comparison of treated and control parasites, there was no clear difference or a slight increase in the pattern of K48-linked ubiquitylation, but the level of K63-linked ubiquitylation was clearly diminished in the rapamycin-treated cells. In the control-treated cell lysate, six discrete bands were clearly visible, with molecular masses in the range of 32 to ~ 150 kDa and the intensity of at least five of these was reduced. These results indicate that the putative UBC13 is largely responsible for synthesis of K63-linked ubiquitin ubiquitylation in blood stage parasites of *P. falciparum*, and suggest that the PF3D7_0527100 gene product is a functional homologue of UBC13, which is responsible for K63-linked ubiquitylation in human cells.

### *ubc13* deletion increases parasite sensitivity to DNA damage induced by treatment with methyl methanesulfonate

**Figure 3:**
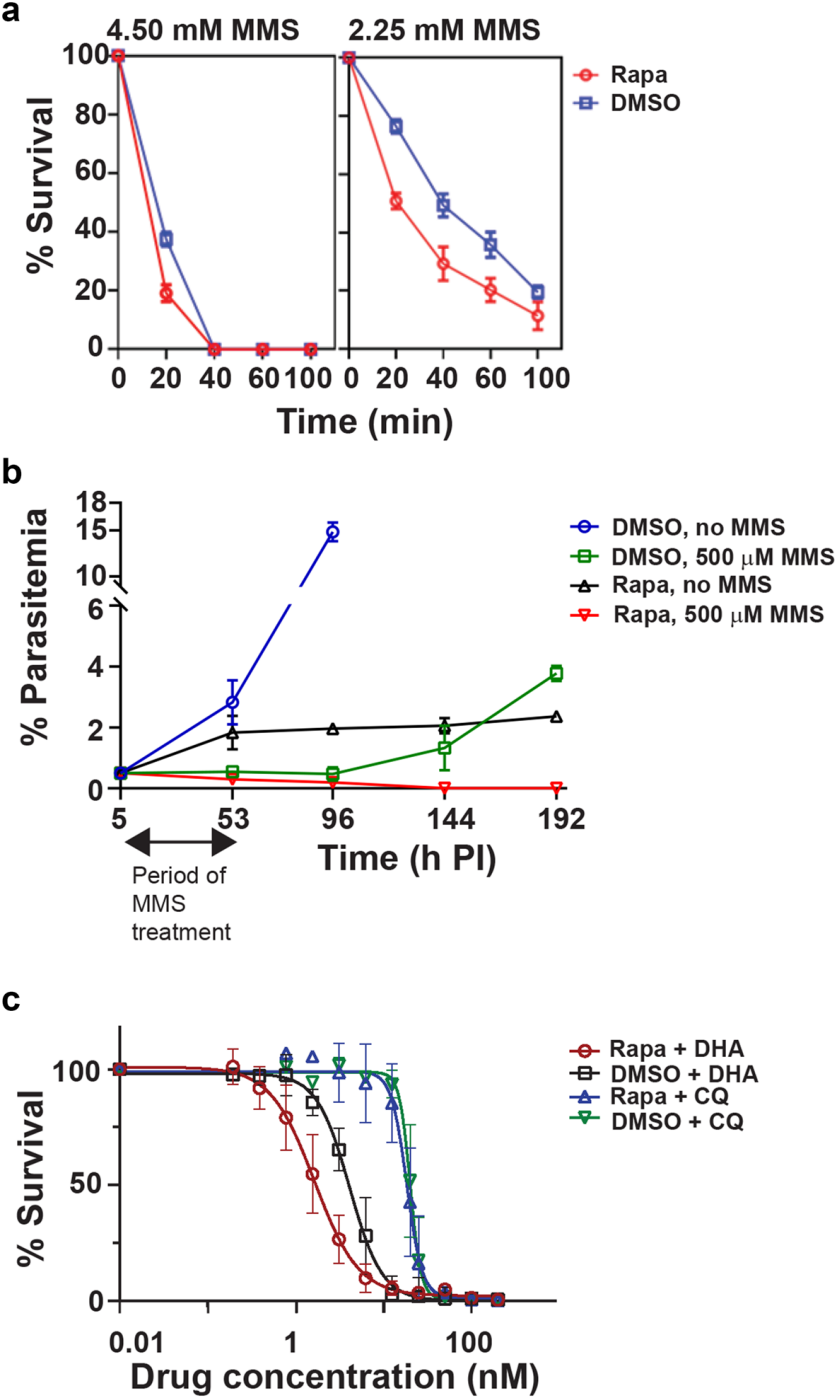
Deletion of *Pfubc13* renders parasites more susceptible to the mutagen, methyl methanesulfonate (MMS) and dihydroartemisinin (DHA), but not chloroquine (CQ). (**a**) Synchronised parasite populations (5 to 8 h post invasion [PI], 1% parasitemia and 3% hematocrit) were pre-treated with DMSO (control) or rapamycin to induce *ubc13* excision for 24 h, and then treated with 2.25 mM or 4.5 mM MMS for 0, 20, 40, 60 and 100 min. Parasite survival was assessed at 48 h. The data shown are mean ± SEM from duplicate experiments. (**b**) Long term survival of parasites treated with MMS. Synchronized parasite populations (5 h PI, 0.5% parasitemia and 3% hematocrit) were treated with rapamycin or DMSO and with or without 500 µM MMS. The cells were then washed to remove the drugs 48 h after the start of the experiment and the incubation was continued in complete medium. Giemsa-stained thin blood smears were examined at the time points indicated to determine percentage parasitemia. The data shown are mean ± SEM from triplicate experiments. (**c**) Parasites were treated with rapamycin or DMSO and increasing concentrations of either DHA or CQ. The percentage parasite survival was measured, allowing the EC_50_ of the drugs in each condition to be calculated. The data shown are mean ± SEM from triplicate experiments The DHA EC_50_ was 1.69 nM (95% CI: 1.41 to 1.99) and 4.1 nM (95% CI: 3.63 to 4.63) for the rapamycin and DMSO treated parasites, respectively, which is significantly different (P < 0.005, t-test), whereas there was no significant difference in the CQ EC_50_ (18.51 [95% CI: 16.05 to 20.20] and 20.19 [95% CI: 18.53 to 21.48]) for the two treatments.

In human cells UBC13 plays an important role in DNA damage repair^19,22^ and we were interested to examine whether this protein has a similar function in *P. falciparum.* Therefore, we induced DNA damage in the parasite following rapamycin treatment, by incubation with MMS, a mutagen that alkylates DNA bases and induces single and double-strand breaks, and which has been shown to cause DNA damage in *P. falciparum*^30,33^. Parasites were exposed for different periods to either 2.25 mM or 4.5 mM MMS, washed to remove the drug and then cultured further to 48 h after initial invasion, at which point parasite survival was measured. The rapamycin-induced *ubc13-iKO* parasite was more sensitive to MMS than the DMSO-treated parasite. This was particularly evident at 2.25 mM MMS, since at the higher MMS concentration 40 min or more exposure resulted in no survival of either parasite population (Fig. 3a).

The effect of adding rapamycin and a lower concentration of MMS together in the first intracellular cycle was also investigated. Parasites were treated with and without 20 nM rapamycin and with and without 500 µM MMS for 48 h and then parasitemia was monitored for up to four cycles of growth. The parasites not treated with rapamycin were severely inhibited by MMS but started to recover in cycle 3 at 144 h. In contrast the rapamycin and MMS treated parasites were killed and no recovery over the period of the experiment was observed (Fig. 3b).

### *Pfubc13* deletion increases parasite sensitivity to dihydroartemisin (DHA) but not chloroquine (CQ)

DHA generates oxidative free radicals that can cause DNA damage^30^. In contrast, CQ is not known to act in this way. Therefore, we examined parasite sensitivity to DHA and CQ, in mock treated or rapamycin-treated *ubc13* inducible knockout parasites. The results (Fig. 3c) revealed that the *ubc13*-iKO parasite is more sensitive to DHA than the control parasite, with a significantly decreased DHA IC_50_ (1.69 nM and 4.1 nM for the rapamycin and DMSO treated parasites; P < 0.005). In contrast, the CQ IC_50_ was not affected by *ubc13* deletion (18.51 and 20.19 for the two treatments). These results suggest that PfUBC13 functions, at least in part, in the DNA damage repair system.

### Treatment of parasites with NSC697923 mimics the induced *ubc13* knockout

**Figure 4:**
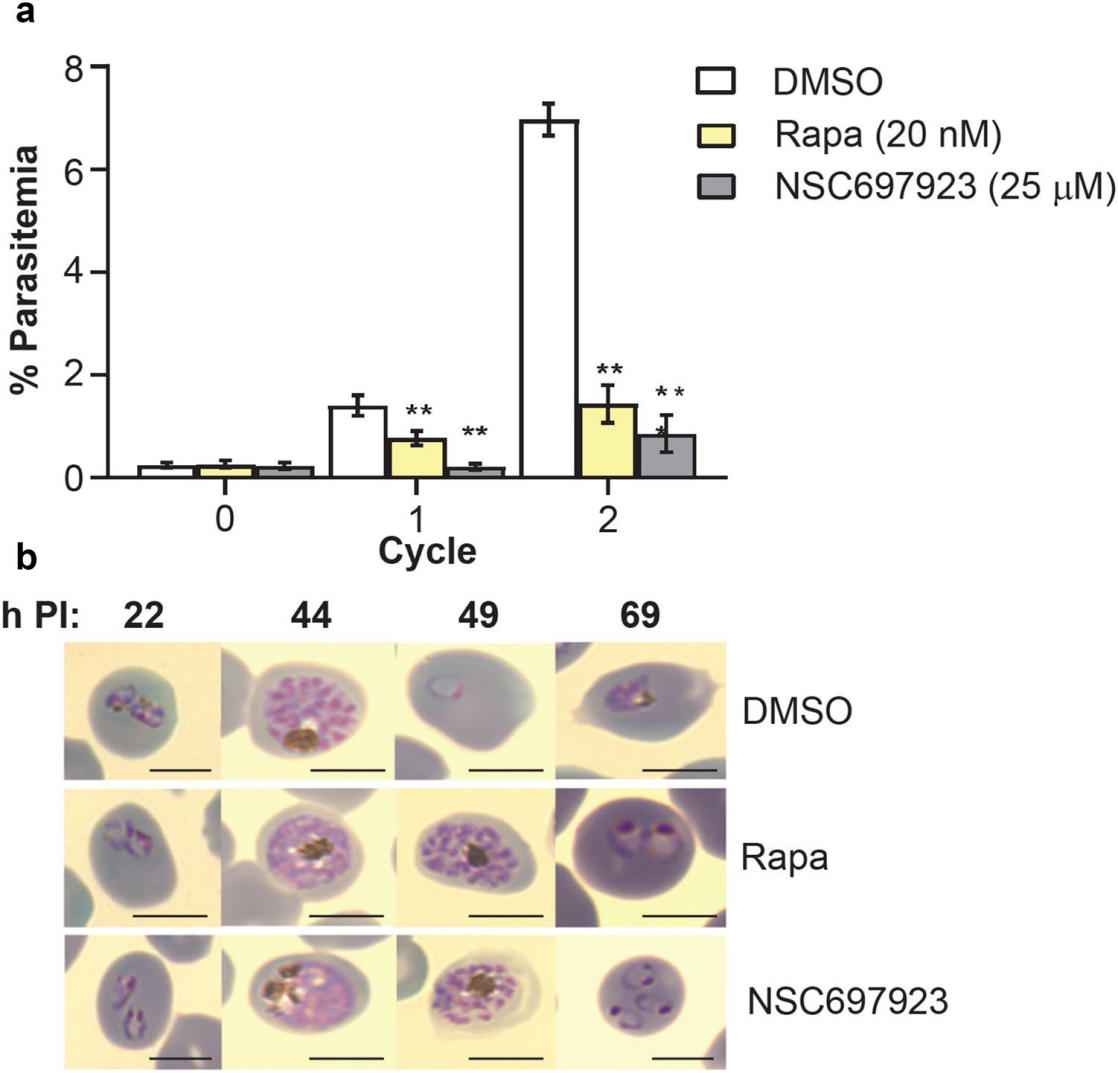
Both NSC697923 and rapamycin treatment affect parasite growth. (**a**) Parasite growth over two cycles of intraerythrocytic development for DMSO-, rapamycin- or NSC697923-treated parasites. Synchronised parasite populations (0.2% parasitemia) were treated with either DMSO (control), rapamycin (to induce excision within *ubc13*) or NSC697923 (to inhibit UBC13), and parasitemia was measured for two cycles by FACS analysis. The data shown are mean ± SEM from triplicate experiments. The parasitemia in both rapamycin and NSC697923-treated parasite culture was significantly lower than that of the control at both cycle 1 and cycle 2. (** p < 0.001) (**b**) Analysis by microscopy of Giemsa-stained parasites at 24, 44, 49 and 69 h post-invasion. All parasites develop to schizonts but further development of both rapamycin- and NSC697923-treated parasites is delayed: at 49 and 69 h PI control parasites have formed new ring stage and trophozoite stages, whereas both rapamycin and NSC697923-treated parasites are largely schizont and early ring stages at these two time points. Scale bar is 5 µm.

NSC697937 is a UBC13 inhibitor in human cells and forms a covalent adduct with the active site cysteine^28^. Since NSC697937 has been shown to inhibit the asexual blood stage development of *P. falciparum* ^29^, we wished to compare the outcome of rapamycin and NSC697923 treatment on the growth of blood stage parasites. Assessment of the percent parasitemia by FACS at the start of the experiment (2 h post invasion) and at time points corresponding to the first cycle (50 hpi) and second cycle (98 hpi) showed that the treated parasites showed significantly reduced parasite numbers (Fig. 4a; DMSO treatment compared to either rapamycin or NSC697937 treatments, p < 0.0001). Giemsa stained thin blood smears were prepared to examine parasite development and morphology after treatment. Both NSC697923 and rapamycin treatment resulted in a growth defect. Both treatments resulted in slowed growth; for example, at 49 h post-invasion, treated parasites with either treatment were largely at the schizont stage, whereas the control parasites were predominantly at the ring stage (Fig. 4b).

## Discussion

Human UBC13 (HsUBC13) specifically catalyzes the formation of K63-linked polyubiquitin chains, which are important in, for example, cytoplasmic NF-κB signaling and the DNA repair system. In *P. falciparum*, the role of UBC13 is unclear although PF3D7_0527100 appears to be an essential gene^26^ and the protein’s function is controlled by the activity of PK9^24,27^. In this study, we generated a transgenic parasite to allow the inducible knockout of this gene using the DiCre recombinase strategy, and examined the resultant phenotype.

The structural alignment of the PfUBC13 and HsUBC13, and the fact that deletion of *Pfubc13* affected K63-linked ubiquitylation in the parasite, indicate that the protein’s catalytic function mirrors that of UBC13 in higher eukaryotes. These data, together with crystal structure information for PfUBC13 (https://www.rcsb.org/structure/2r0j) and PfUBC13 complexed with PfUEV1a (https://www.rcsb.org/structure/3e95) indicate that PF3D7_0527100 is a homolog of UBC13 in higher eukaryotes. This is the first demonstration of the potential role of PfUBC13 in K63-linked polyubiquitylation in the parasite because the previous studies focused on PK9 and PK9 inhibitors^24,27^. The six visible K63 ubiquitylated substrates ranged in size from 32 to ~ 150 kDa with no evidence of heterogeneity, suggesting that the ubiquitylation is either a chain of just two or a small discrete number of ubiquitin units. These substrates remain to be identified, although they are likely present in the experimentally determined trophozoite/schizont ubiquitomes^29^. Interestingly, K63-polyubiquitylation of histone H2A^34^ and proliferating cell nuclear antigen (PCNA)^35^ is important in DNA repair processes, and both these proteins are present in the *P. falciparum* ubiquitome. In future work it may be possible to identify the proteins directly following their affinity purification with K63 polyubiquitin-specific antibody. Their identification may illuminate further the biological processes in which this modification is involved in the parasite. The slight increase in K48 ubiquitylation in rapamycin treated cells may reflect a loss of proteostasis, leading to enhanced ubiquitylation of proteins destined for degradation in the proteasome.

Rapamycin-induced *Pfubc13* deletion affected parasite growth severely. After one cycle the number of new ring stage parasites was reduced significantly but not completely; and this effect was amplified at the second cycle and beyond, although morphologically intact parasites were still present in the culture at cycle 5. These parasites did not have an intact *ubc13* gene and some visible abnormalities were present in late stages, such as pyknotic and deformed cells. Some parasites could develop from ring to schizont stage, and apparently survive to the next cycle although growth was severely retarded. Parasite largely failed to develop into viable late stages, which is when DNA replication, nuclear division and merozoite differentiation occur, suggesting a role of Pfubc13 in maintaining normal DNA replication.

K63-linked polyubiquitylation has been identified to play a role in DNA repair, signal transduction, and kinase activation in eukaryotes^5,6,34^, while K48-linked polyubiquitylation targets protein substrates to the proteasome. Therefore, we chose to examine the importance of PfUBC13 in DNA repair processes for *P. falciparum*. In *Plasmodium spp*., DNA damage has been studied experimentally and can result from exposure to UV irradiation; ionizing radiation like X-rays and gamma rays; chemical mutagens such as alkylating agents (e.g., methyl methanesulfonate and cisplatin) and also exposure to oxidative free radicals^33,36^. The damage includes inter-strand cross-linking, DNA strand breaks, and inhibition of DNA synthesis and replication, leading to cell cycle arrest, and parasite death^37^. Genome integrity is maintained in the parasite by repair mechanisms, for example, homologous recombinant repair (HR), mismatch repair (MMR), nucleotide excision repair (NER) and base excision repair (BER). Canonical non-homologous end joining (C-NHEJ) repair is absent in *Plasmodium*, but an alternative inefficient NHEJ pathway has been described^38,39^.

We chose to use the DNA alkylating agent, MMS, which predominantly modifies guanine to N7-methyl guanine (7meG) and O6-methyl guanine (O6meG), to study the effect of *ubc13* deletion on DNA repair. These lesions can lead to the collapse of the replication fork and subsequent induction of DNA double-strand breaks^40^ that are commonly repaired by BER, NER and MMR processes^37,41^. Up-regulation of genes that function directly in DNA repair mechanisms such as PfRAD51 and PfRAD54, and changes in histone modification were reported after MMS treatment of *P. falciparum*^30,42^. These changes in chromatin modulate the access of repair factors to the damage site resulting in DNA repair^43^. The up-regulation of *Pfubc13* transcription in the MMS-treated parasite has not been reported^30^. However, our results show that the *Pfubc13-*inducible knockout parasite has greater sensitivity to MMS treatment, and fails to survive under conditions in which the intact parasite recovers. These results are consistent with a major role for UBC13 and K63-polyubiquitylation in DNA repair in *P. falciparum*.

DHA is the active metabolite of artemisinin and related compounds, and its mode of action against the malaria parasite is complex^44,45^. One property, following activation, is its ability to alkylate macromolecules, and recent studies have suggested that one cellular process where this is important is in DNA damage and repair^30,31^. The *Pfubc13*-inducible knockout parasite had increased sensitivity to DHA, with a significantly decreased IC_50_, and showing a similar response profile to that with MMS, while CQ did not show this effect. This suggests that DHA is acting at least in part through mechanisms where repair is dependent on UBC13 and K63 ubiquitylation.

NSC697923 is an inhibitor of HsUBC13 function, reacting with the sulfhydryl group of the active site Cys87 to form a 5-nitrofuran adduct^28^. It has activity against *P. falciparum* growth *in vitro*^29^, and therefore it was of interest to compare mock and rapamycin-treated *Pfubc13*- inducible knockout parasites to parasites treated with this drug. Both treatments produced a similar result with a similar delay in parasite development. Although NSC697923 is not a potent inhibitor, the results suggest that if the primary target of NSC697923 in the parasite is UBC13 then better compounds with higher affinity, specificity, and a good therapeutic index, could be potential antimalarial compounds. Furthermore, we would expect that inhibitors of PfUBC13 could be used in combination to enhance the efficacy of artemisinin drugs.

In conclusion, our results demonstrate that the PF3D7_0527100 gene product is PfUBC13, a functional homologue of HsUBC13, which is responsible for K63-linked ubiquitylation and is involved in DNA repair systems. Deletion of this gene results in a *P. falciparum* growth defect and increases the sensitivity of the parasite to agents that cause DNA damage, in particular MMS and DHA.

## Materials and methods

### Bioinformatics

HsUBC13 and PfUBC13 sequences were obtained from the Uniprot (https://www.uniprot.org/uniprot/P61088) and PlasmoDB (https://plasmodb.org/plasmo/app/record/gene/PF3D7_0527100) databases, respectively. The amino acid sequences were aligned using the Protein BLAST alignment tool (https://blast.ncbi.nlm.nih.gov). Three-dimensional structures were generated with Phyre2^46^.

### In vitro culture and synchronisation of *P. falciparum*

The inducible DiCre recombinase-expressing *P. falciparum* line II-3^32^ was cultured in RPMI 1640 medium supplemented with 1% (w/v) Albumax at 37˚C in gassed (5% CO_2_, 5% O_2_, 90% N_2_) flasks. The parasite population was synchronized by schizont centrifugation onto a 63% Percoll cushion. Purified schizonts were cultured for 2 to 3 h to allow merozoite invasion of fresh red blood cells and ring stage formation, and then residual schizonts were lysed by treatment with 5% D-sorbitol for 10 min. RBCs were purchased from the United Kingdom National Health Service Blood and Transplant (NHSBT).

### Generation of an inducible *Pfubc13* knockout parasite

To generate an inducible knockout of *ubc13*, we used CRISPR-Cas9 to insert two loxP sites flanking the last exon of the gene and the rapamycin-inducible active DiCre-recombinase system to excise the intervening DNA so that a truncated inactive protein would be produced, using the methodology described previously^29,32^. The Guide RNA sequence was designed to direct Cas9 to introduce a double-stranded break in Exon IV (Supplementary Fig. 1 and Supplementary Table 1). A pair of complementary oligonucleotides was phosphorylated with T4 polynucleotide kinase, annealed, and ligate into the plasmid pDC2-Cas9-hDHFRyFCU that had been digested with BbsI restriction enzyme^32^. For the repair plasmid, synthetic DNA (GeneArt, Life Technologies) was used to replace Intron IV with a synthetic intron containing a 34 bp loxP element (loxPint, 103 bp)^47^ followed by a recodonized Exon IV (225 bp) and a second loxP site (34 bp) after the stop codon. This sequence was placed between two homology regions, HR1 (262 bp) and HR2 (352 bp) to facilitate homologous recombination and cloned in the pMK vector with EcoRI and XhoI restriction sites at the 5’ and 3’ end, respectively. The repair plasmid (50 μg) was digested with EcoRI and XhoI, ethanol precipitated together with 20 μg plasmid to express the guide RNA, and redissolved in 10 μl sterile TE (10 mM Tris–HCl 1 mM EDTA pH 8.0).

For transfection, purified *P. falciparum* II-3 schizonts (10–40 µl) were mixed with DNA dissolved in 100 µl of AMAXA primary cell solution P3 and electroporated using an AMAXA 4D nucleofector with program FP158. Following electroporation, schizonts were transferred to 2 ml culture medium containing 300 µl of erythrocytes, and incubated with shaking at 37 °C for 30 min. A further 8 ml of culture medium was added and then 24 to 48 h post-transfection at 37 °C, 2.5 to 10 nM WR99210 was added to provide selection. The parasite population was screened for integration by PCR, using pairs of primers (Supplementary Fig. 1 and Supplementary Table 1) and then individual lines were selected following cloning by limiting dilution and screening by PCR.

### Rapamycin-induced DiCre mediated loxP excision

Synchronized ring-stage parasites (2 h PI) were treated with a final concentration of 20 nM rapamycin or with DMSO for 24 h. Samples were taken for DNA analysis by PCR at this time or for protein analysis by Western blotting at 44 h PI.

### Parasite growth and survival study

One hundred microliters synchronised ring-stage parasites (2 h PI, 0.2% parasitemia; 3% hematocrit), treated with 20 nM rapamycin or 0.01% DMSO, were placed in wells of a round-bottom 96-well plate at time zero. At the start and after specific periods (48 and 96 h) under normal culture conditions, parasite samples were fixed with 100 µl 4% paraformaldehyde + 0.1% glutaraldehyde for 1 h at room temperature (RT). The plates were centrifuged and fixative was replaced with 50 µl PBS and the parasites were stained with 2 × SyBR Green I in the dark for 30 min. One microlitre of stained cells was diluted into 3 ml PBS in a FACS tube and analysed using a BD LSRFortessa flow cytometer, with a 488 nm UV laser and fluorescence at 530 nm. Duplicate samples on triplicate plates were analysed for each time point. Data were analysed with one-way ANOVA analysis (Dunnett’s multiple comparison test) using Graphpad software. At various time points thin blood smears were prepared, stained with Giemsa’s reagent and examined by microscopy to evaluate parasitemia and parasite morphology.

### Western blotting of parasite lysates

Parasites (3–4 h PI) were treated with 20 nM rapamycin or 0.01% DMSO for 40 h, then schizonts were purified by centrifugation over a 63% Percoll cushion, suspended in PBS containing 0.15% (w/v) saponin to lyse erythrocyte membranes, and collected by centrifugation at 5,000 g for 5 min. The cell pellet was dissolved in 10 volumes of lysis buffer (150 mM NaCl, 1% Triton X-100, 0.1% SDS, 1 μl/ml benzonase [Roche], and 1X complete protease inhibitors [Roche]). After incubation on ice for 20 min, samples were centrifuged at 17,000 g for 30 min. The protein content of the supernatant was measured using a BCA Protein Assay (Pierce), and 10 μg total protein was resolved on a 4 to 12% Tris–acetate PAGE gel (Invitrogen), transferred to nitrocellulose, and probed with specific antibodies. Protein ubiquitylation was detected with antibodies to ubiquitin Lys48- and Lys63-specific linkages (Merck-Millipore rabbit mAbs 05–1307 and 05–1308, respectively). Antibodies to BiP^29^ were used as a loading control.

### Parasite survival following treatment with the mutagen, methyl methanesulfonate

Parasites pretreated with rapamycin or DMSO for 24 h were treated with 2.25 mM and 4.5 mM MMS for 0, 20, 40, 60 and 100 min, washed twice with incomplete RPMI 1640 medium and finally returned to culture in complete RPMI 1640 medium. At 48 h after the start of the experiment, thin blood smears were prepared, stained with Giemsa’s reagent and examined by microscopy to calculate percent parasite survival.

To examine parasite recovery from MMS induced-DNA damaged, ring stage parasites 4 to 6 h PI were adjusted to 0.5% parasitemia, 3% hematocrit and then treated for 48 h with 20 nM rapamycin or DMSO and with or without 500 μM MMS. The medium was changed every 2 days, and the percentage parasitemia calculated every day from examination of Giemsa-stained thin blood smears.

### Parasite drug sensitivity

Synchronised ring stage parasites (0.05–1% parasitemia; 3% hematocrit) were treated with either DMSO alone or 20 nM rapamycin, together with serial dilutions (from 200 nM) of DHA and CQ as 100 µl final volume of culture in a black 96-well plate. Following incubation under normal culture conditions for 48 or 96 h, parasite survival was determined by using the malaria SYBR Green I-based fluorescence assay (MSF), as described previously^48^. Briefly, 100 µl of SYBR Green I in lysis buffer (0.2 µl of SYBR Green I/ml of lysis buffer [20 mM Tris–HCl, 5 mM EDTA, 0.008% saponin, and 0.08% Triton X100]) was added to each well, then the plate was mixed and incubated in the dark at RT for 1 h. The fluorescence was measured with excitation and emission at 485 and 530 nm, respectively^48–50^. The data were analysed and the EC_50_ for each compound was calculated using GraphPad Prism software.

## Supplementary Information

Supplementary Information.

## Data Availability

All plasmids and transgenic parasites described in this study are available upon request.
