# Supplementary material for: Deletion of Plasmodium falciparum ubc13 increases parasite sensitivity to the mutagen, methyl methanesulfonate and dihydroartemisinin
Source: Sci Rep. 2021 Nov 8;11:21791. doi: 10.1038/s41598-021-01267-6 (PMC8575778; doi:10.1038/s41598-021-01267-6)

Deletion of *Plasmodium falciparum ubc13* increases parasite sensitivity to the mutagen, methyl methanesulfonate and dihydroartemisinin

Supawadee Maneekesorn[1,2], Ellen Knuepfer[2+], Judith L. Green[2], Parichat Prommana[3], Chairat Uthaipibull[3#], Somdet Srichairatanakool[1] and Anthony A. Holder[2*]

[1]Department of Biochemistry, Faculty of Medicine, Chiang Mai University, Chiang Mai 50200, Thailand; [2]Malaria Parasitology Laboratory, The Francis Crick Institute, 1 Midland Road, London, NW1 1AT, United Kingdom; [3]Medical Molecular Biotechnology Research Group, National Center for Genetic Engineering and Biotechnology (BIOTEC), 113 Thailand Science Park, Phahonyothin Road, Khlong Nueng, Khlong Luang, Pathum Thani 12120, Thailand.

Supplementary material

Supplementary Table 1: Oligonucleotides used to generate guide RNA or for primers in PCR analysis of parasite genetic modification.

Oligonucleotide	Sequence
Guide A	ATTGTCAGCAACTTTCGAATCTAA
Guide B	AAACTTAGATTCGAAAGTTGCTGA
P1	GAAACACAAAACTTAGCGAATGAACCACG
P2	GCTTGTATGCTTAAGAGTACTGTACG
P3	GATGCCGAACATGTTGCGAGACAATG
P4	GTGCGCAAAAAGATATAATTTTAATATTAAGAC
P5	CCCGTCAATGGAACAAGATATACGCC
P6	CCTTAAGAATGTCTAGGCATATACGTC

Supplementary Figure 1: Construction of a floxed *Pfubc13* locus in the *P. falciparum* II-3 genome, and rapamycin induced Di-Cre mediated excision. a. CRISPRCas9 was used to introduce a DNA double-strand break in the *ubc13* locus, using a guide sequence located in the fourth exon. Exon IV also includes the codon for the active site cysteine (C). The repair plasmid contained HR1 and HR2, two regions of homology to the gene, flanking a synthetic sequence for intron IV containing a loxP element, followed by a recodonised exon IV and a second loxP site. The active site cysteine is coded by the seventh codon in exon IV. Repair by homologous recombination within the homology regions HR1 and HR2 produces a modified locus. **b.** Integration and modification of the parasite DNA was monitored by PCR amplification using primer pairs designed to yield products specific for either the unmodified or the modified locus. The locations of primer pairs and the expected size of PCR products are indicated. Primer pairs P1/P2 and P3/P4 were expected to produce a product only from the

unmodified locus and P1/P6 and P5/P4 were expected to produce a product only from the modified locus. **c.** PCR analysis of the parasite population following transfection, the parental line and a cloned parasite line (2E). The uncloned population contains both recombinant and parental parasites, whereas clone 2E has only the modified gene locus (*ubc13.loxP*) since amplification from the P1/P6, and P5/P4 primers pairs and the lack of product from the P1/P2 and P3/P4 primer pairs are diagnostic for correct integration of the modified DNA. **d.** The *ubc13.loxP* locus before and after DiCre mediated excision induced by rapamycin treatment. The location of primer pair P1/P4 and the expected sizes of products resulting from PCR amplification of DNA from either treated or untreated parasites are indicated. **e.** PCR amplification with primers P1/P4 from the 2E parasite clone after treatment with either DMSO or rapamycin for 24 h. Full length gels are shown in Supplementary Fig. 5.

Supplementary Figure 2: Parasite morphology in the cycles of growth following treatment with rapamycin to truncate the *ubc13* gene or with DMSO (control). At cycle 1, 2 and 5, thin smears of parasitized RBC were Giemsa-stained and examined by light microscopy.

Supplementary Figure 3: Full length agarose gels for the PCR analysis of cycle 3 and cycle 5 parasites. This figure relates to Fig. 2 panel d.

Supplementary Figure 4: Immunoblot analysis of schizont lysates at three different exposure times. This figure relates to Fig. 2 panel e.

Supplementary Figure 5: Full length agarose gels for the PCR analysis of modified and parental parasite populations, a cloned modified parasite, and the rapamycin-induced DiCre-mediated excision of *ubc13*. This figure relates to Supplementary Fig. 1 panels c and e.

Supplementary Fig. 1

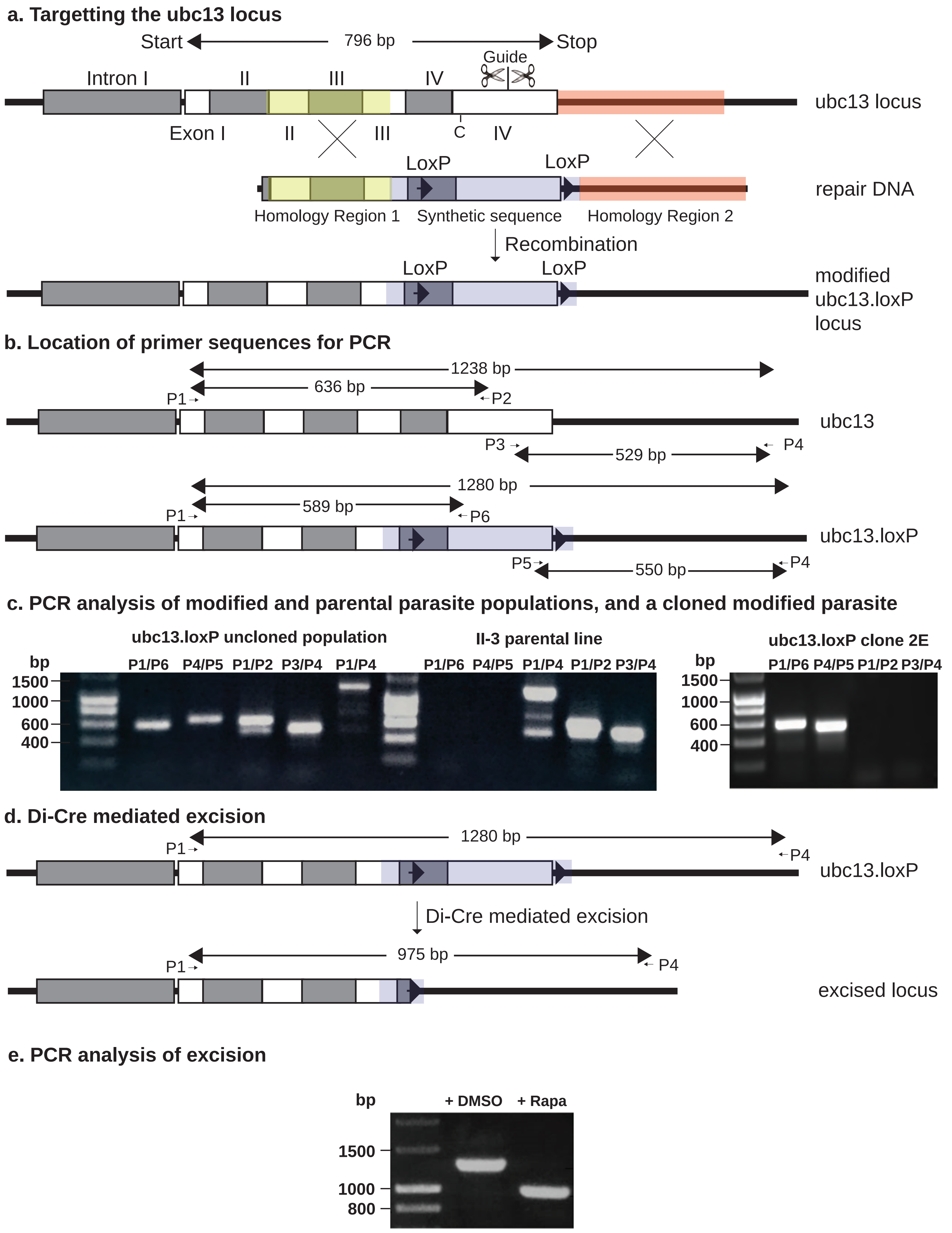

Supplementary Fig. 2

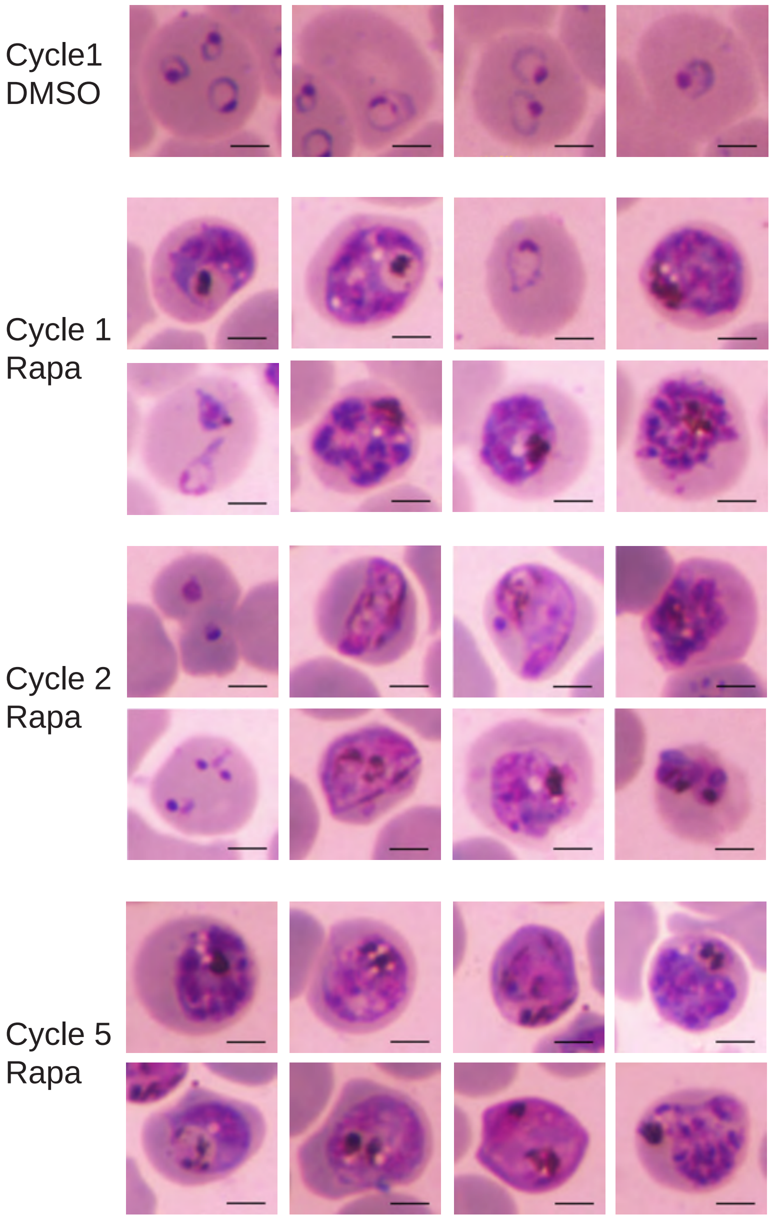

Supplementary Fig. 3

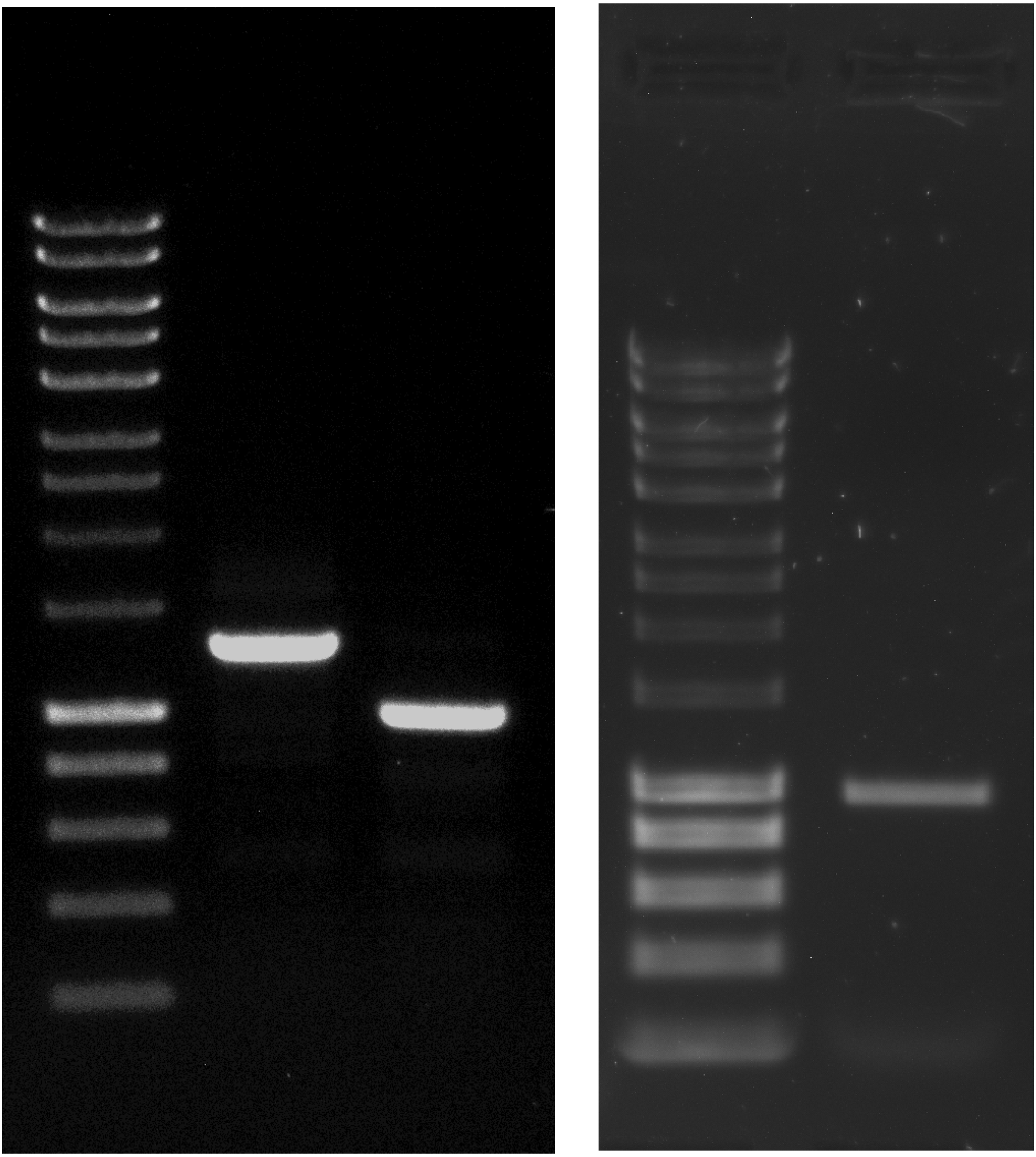

Supplementary Fig. 4

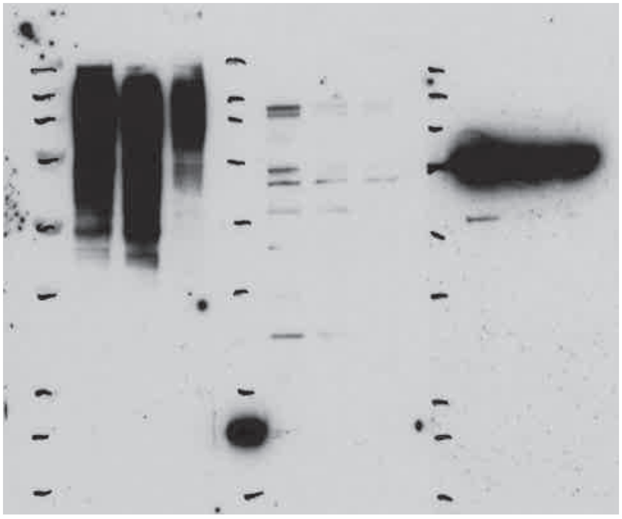

3 min exposure

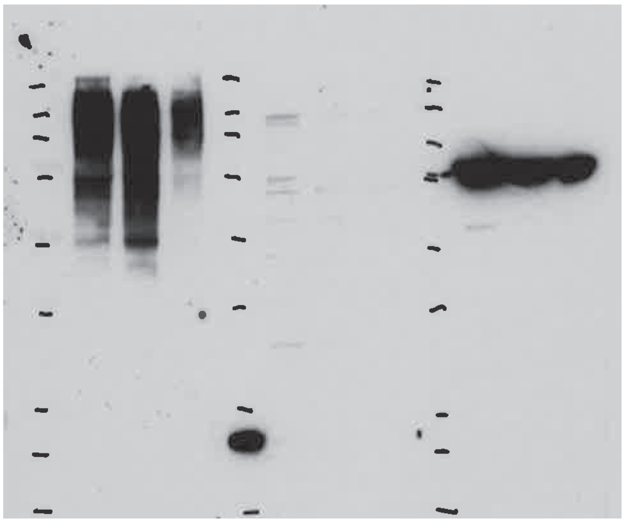

30 sec exposure

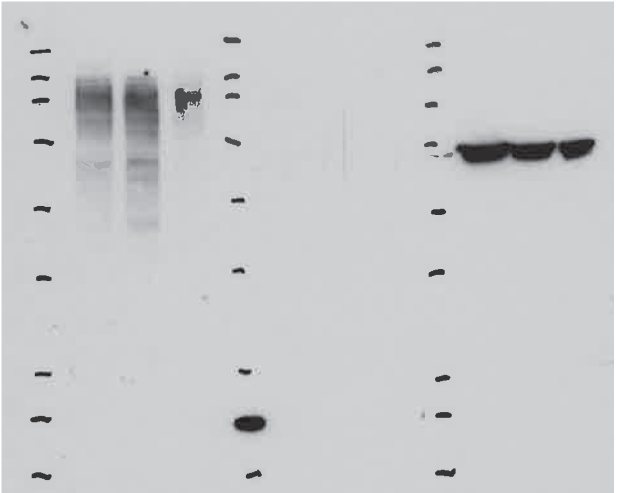

5 sec exposure

Supplementary Fig. 5

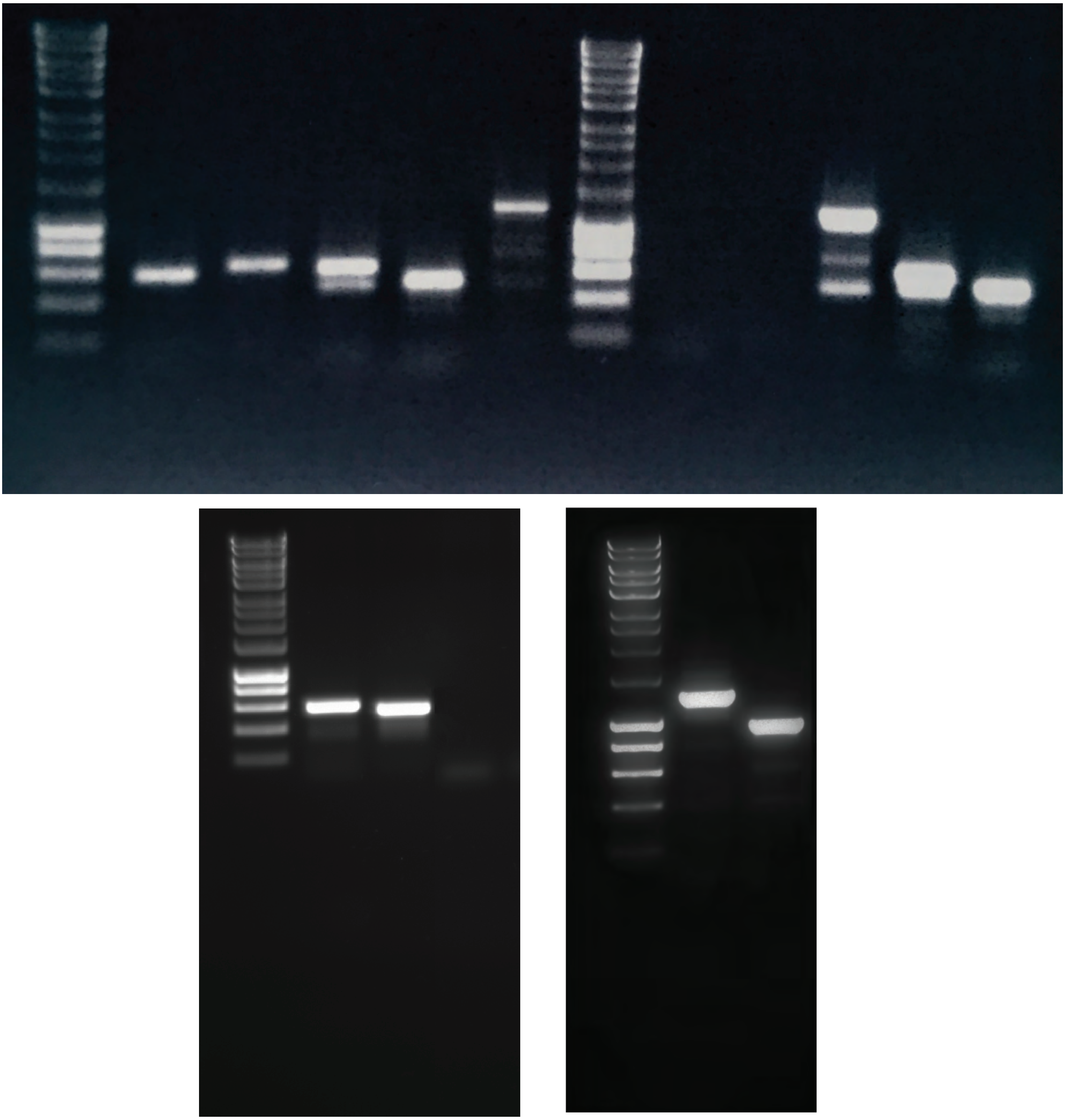

Supplement: Supplementary file 1 — Supplementary Information. [file 41598_2021_1267_MOESM1_ESM.pdf]
